# Increased accumulation of magnetic nanoparticles by magnetizable implant materials for the treatment of implant-associated complications

**DOI:** 10.1186/1477-3155-11-34

**Published:** 2013-10-10

**Authors:** Nina Angrisani, Franziska Foth, Manfred Kietzmann, Stephan Schumacher, Gian Luigi Angrisani, Anne Christel, Peter Behrens, Janin Reifenrath

**Affiliations:** 1 Small Animal Clinic, University of Veterinary Medicine, Foundation, Bünteweg 9, 30559 Hannover, Germany; 2 Institute of Pharmacology, Toxicology and Pharmacy, University of Veterinary, Medicine, Foundation, Bünteweg 17, 30559 Hannover, Germany; 3 Institute of Material Sciences, Leibniz University Hannover, An der Universität 2, 30823 Garbsen, Germany; 4 Institute of Inorganic Chemistry, Leibniz University Hannover, Callinstraße 9, 30167 Hannover, Germany

**Keywords:** Implant directed magnetic drug targeting, Martensitic steel, *In vitro*, Magnetic field strength

## Abstract

**Background:**

In orthopaedic surgery, accumulation of agents such as anti-infectives in the bone as target tissue is difficult. The use of magnetic nanoparticles (MNPs) as carriers principally enables their accumulation via an externally applied magnetic field. Magnetizable implants are principally able to increase the strength of an externally applied magnetic field to reach also deep-seated parts in the body. Therefore, the integration of bone-addressed therapeutics in MNPs and their accumulation at a magnetic orthopaedic implant could improve the treatment of implant related infections. In this study a martensitic steel platelet as implant placeholder was used to examine its accumulation and retention capacity of MNPs in an *in vitro* experimental set up considering different experimental frame conditions as magnet quantity and distance to each other, implant thickness and flow velocity.

**Results:**

The magnetic field strength increased to approximately 112% when a martensitic stainless steel platelet was located between the magnet poles. Therewith a significantly higher amount of magnetic nanoparticles could be accumulated in the area of the platelet compared to the sole magnetic field. During flushing of the tube system mimicking the *in vivo* blood flow, the magnetized platelet was able to retain a higher amount of MNPs without an external magnetic field compared to the set up with no mounted platelet during flushing of the system. Generally, a higher flow velocity led to lower amounts of accumulated MNPs. A higher quantity of magnets and a lower distance between magnets led to a higher magnetic field strength. Albeit not significantly the magnetic field strength tended to increase with thicker platelets.

**Conclusion:**

A martensitic steel platelet significantly improved the attachment of magnetic nanoparticles in an *in vitro* flow system and therewith indicates the potential of magnetic implant materials in orthopaedic surgery. The use of a remanent magnetic implant material could improve the efficiency of capturing MNPs especially when the external magnetic field is turned off thus facilitating and prolonging the effect. In this way higher drug levels in the target area might be attained resulting in lower inconveniences for the patient.

## Background

Implant related infections as well as non- or poorly-healing fractures still issue a challenge to the physician in charge. Between 2003 and 2009, about 1.38 million hip prostheses and 1.01 million knee prostheses have been implanted [1]. Depending on predisposing factors 1-7% develop implant-related infections [2,3]. This rate even rises to 13.9% after surgically fixed open fractures [4]. To avoid infections, systemically administered antibiotics [5] are most commonly used. Besides, antibiotic chains [6] or antibiotic implant coatings [3] are applied as an approach of a close-to-implant administration. However, the general necessity and adequacy is questionable as a prophylactic perioperative antimicrobial treatment cannot be a long-term solution in respect of increasing antibiotic resistance.

Moreover, selective treatment of acute inflammatory processes still remains difficult mainly due to insufficient agent accumulation in the peri-implant area after systemic administration. Thus, excessive drug levels may be necessary to reach adequate concentrations within the target tissue. Therewith the risk of undesirable side effects increases markedly while, however, in many cases success of the treatment is not achieved [7].

The use of magnetic nanoparticles (MNPs) as carriers principally enables the accumulation of either pharmaceuticals (e.g. chemotherapeutics, antibodies, peptide therapeutics) or oligonucleotides and growth factors via external applied magnetic fields in selective areas of the organism. For this purpose different types of MNPs have been developed in recent years which have properties like low toxicity, adequate circulation times or controllable magnetic responsiveness [8]. Most previous studies focussed on their implementation in tumour treatment [9] while another application under research is the transport of genetic material or stem cells via MNP-incorporation [10,11].

Although numerous animal studies were able to proof the mechanism of action of magnetically driven drug delivery during the last 25 years the transfer to clinical use has failed as yet [12]. One of the main reasons for this is the decrease of the magnetic field strength as a function of distance from the generating magnet [12]. However, magnetizable implants are principally able to increase the strength of an externally applied magnetic field. Thus, adequately designed magnetic nanoparticles could be satisfactorily accumulated in the target tissue. Already in 1997, this concept has been reported for non-porous superparamagnetic nanoparticles in the cardiovascular system [13]. Recent studies consider wires, stents and seeds [14]. So far, no work concentrated on magnetic plates for orthopaedic applications. Unfortunately, the accumulation of agents such as anti-resorptives, anti-infectives or anabolics in the bone as target tissue is especially difficult [15]. Therefore, the integration of bone-addressed therapeutics in nanoporous shells around MNPs and their accumulation at the orthopaedic implant would enable the orthopaedist to considerably improve the treatment of the complications described above. Austenitic steel 316 L or titanium alloys are commonly used in orthopaedic surgery. They provide only poor magnetic properties. In contrast ferritic or martensitic stainless steel holds properties like significantly higher permeability and coercivity. While martensitic materials provide lower maximum permeability than ferritic ones, their coercivity excels.

**Figure 1:**
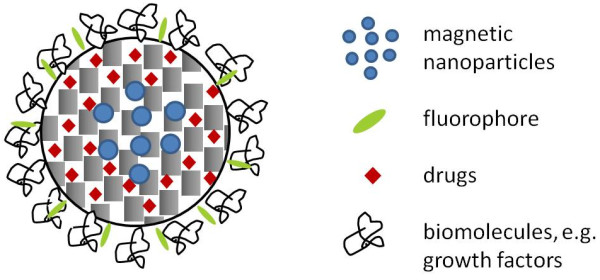
**Magnetic nanoparticles enclosed in a nanoporous silica shell.** The inner pore volume of such particles can be used for the future incorporation of drugs; the outer surface can be employed to bind fluorescent molecules for imaging and biomacromolecules for improving biocompatibility.

The aim of the present study was to examine the suitability of a martensitic steel platelet (Type 1.4122) on the ability to accumulate magnetic nanoparticles in an *in vitro* setup. These particles consist of a magnetic core composed of magnetite (Fe_3_O_4_) enclosed by a nanoporous silica layer [16] which serves for the future incorporation of drugs (Figure 1).

## Results

### Basic experiments

Lower values of *r*_*basic*_ indicate higher amounts of accumulated MNPs. The standard setup parameters for the basic experiments included a flow velocity of 1 mm/s, a platelet thickness of 3 mm with 2×2 magnets mounted at a distance of 1.5 cm. If parameters differed or were added for specific setups values are given at the respective paragraph.

#### Influence of tube

At flow velocities of 1 mm/s and 8 mm/s, sedimented MNPs within the tube were visible to the naked eye. Generally, the concentration of MNPs in the collected sample was lower than in the tube sample. A significant lower concentration of MNPs sedimented in the tube at 8 mm/s (r _basic_ = 0.798 ± 0.072) than with the lower rate of 1 mm/s (r _basic_ = 0.64 ± 0.122).

#### Influence of platelet and platelet thickness

Compared to the influence of the tube alone the non-magnetic 2 mm platelet could not increase the MNP accumulation (r _basic_ = 0.736 ± 0.049). The platelet magnetized to saturation generated a low magnetic field (0.0012 T) and accumulated a significantly higher amount of MNPs (r _basic_ = 0.551 ± 0.074) than the non-magnetic platelet.

Regarding platelet thicknesses of 1 mm, 2 mm and 3 mm in combination with mounted magnets, it seems that the quotient decreases slightly for thicker platelets (r _basic 1 mm_ = 0.12 ± 0.03; r _basic 2 mm_ = 0.11 ± 0.05; r _basic 3 mm_ = 0.11 ± 0.05). However, these differences were not statistically significant.

#### Influence of magnet quantities and magnet distances

**Figure 2:**
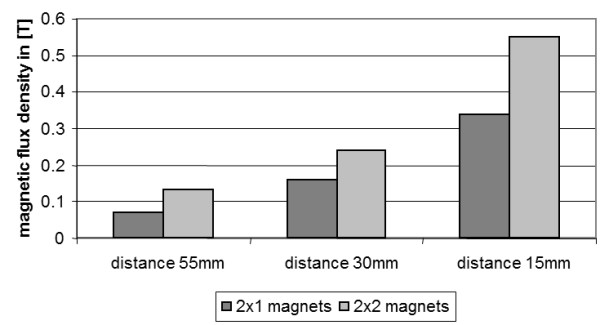
Magnetic flux density in dependence of the number of magnets and the distance between magnets.

As expected, the magnetic flux density generally increased with increasing number of magnets and decreasing distance between the magnets. Therefore the highest magnetic flux density (0.55 T) could be generated by 2×2 magnets at a distance of 15 mm (Figure 2) which resulted in a r _basic_ of 0.134 ± 0.027.

A further increase in magnetic flux density (0.62 T) could be obtained when the 3 mm platelet was additionally mounted. Accordingly, a slightly lower ratio was reached (r _basic 15 mm_ = 0.11 ± 0.05). The r _basic_ values with platelet for 55 mm/0.55 T and 35 mm/0.51 T were 0.25 ± 0.06 and 0.13 ± 0.04, respectively.

**Figure 3:**
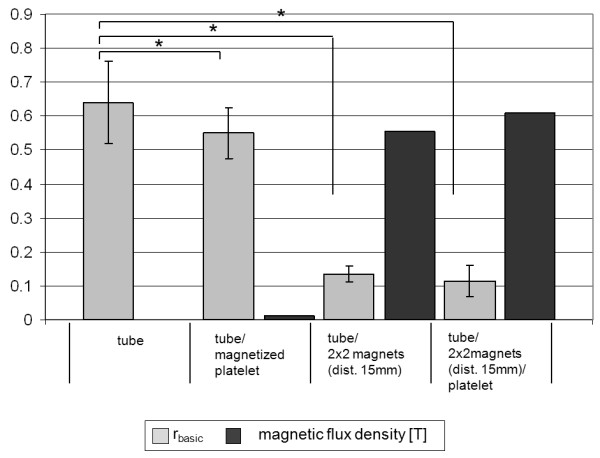
**Influence of tube, magnets and platelet on the accumulation of MNPs.** r _basic_ values (light grey) and magnetic flux density (dark grey) for different experimental setups. The accumulation of MNPs increases with increasing magnetic field strength. Asterisks (*) mark significant results (p < 0.05).

Figure 3 summarizes and compares the results of the predominant experimental setups.

#### Influence of flow velocity

Additionally to the standard flow velocity of 1 mm/s, 2 mm/s, 8 mm/s and 0.5 mm/s were examined.

**Figure 4:**
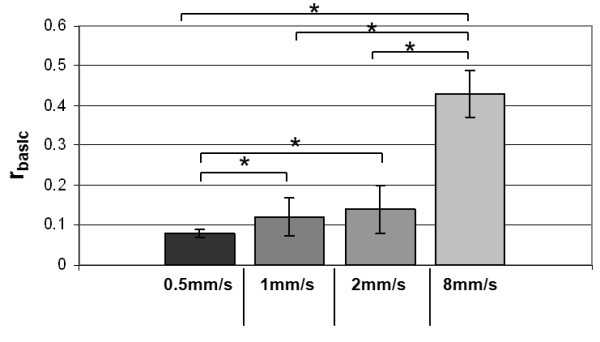
**MNP accumulation at different flow velocities.** The capture capacity is represented by the ratio of the concentrations measured in the collected sample and the tube sample. r _basic_: ratio of basic experiments; c_cs_: concentration of collected sample; c_ts_: concentration of tube sample. All differences except for 1 mm/s vs. 2 mm/s are highly significant (p ≤ 0.01).

In comparison to the influence of the tube alone significantly higher amounts of MNPs accumulated in the tube sample with platelet and 2×2 magnets mounted at a flow velocity of 1 mm/s. Generally, the faster the MNP solution passed through the system, the less MNPs accumulated within the tube (Figure 4, r_basic 8mm/s_ = 0.43 ± 0.06; r_basic2mm/s_ = 0.14 ± 0.02; r_basic0.5mm/s_ = 0.08 ± 0.01).

#### Compartment trial

**Figure 5:**
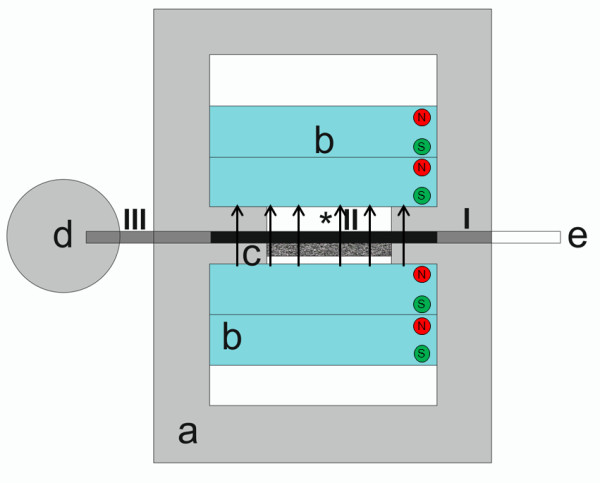
**Compartment trial.** Compartments I-III for determination of the nanoparticle concentration inside the different parts of the tube (**a-e**, *: see Figure 7).

To determinate the quantity of nanoparticles inside the different compartments of the tube system, the volume of three different compartments (I-III, Figure 5) was isolated and examined instead of investigating the content of the complete tube in one.

All compartments showed different quotients. Compartment I (beginning of flow chamber to beginning of magnets) accumulated a lower amount of MNPs (r _basic_ = 0.23 ± 0.09) than compartment II (tube in the region of the magnets; r _basic_ = 0.10 ± 0.04). The lowest amount of MNP accumulation was found for compartment III (r _basic_ = 0.9 ± 0.19). This ratio was significantly higher than in compartment I and II.

### Transfer experiments

Lower values of *r*_*transfer*_ indicate higher amounts of kept MNPs.

*r*_*basic*_ and *r*_*transfer*_ cannot be compared to each other.

#### Retention capacity of tube after MNP accumulation with 2×2 magnets

**Figure 6:**
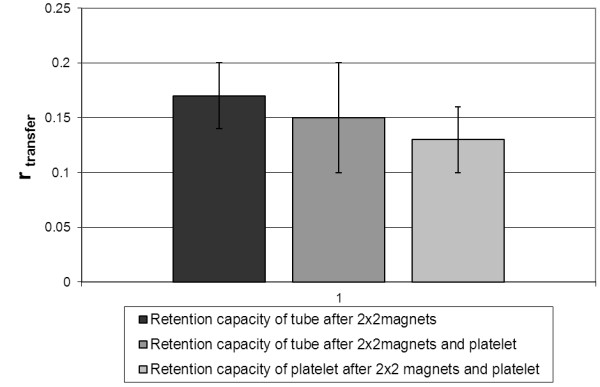
**Flushing experiments simulating the *in vivo *situation.** Retention capacity of the tube or platelet and tube, respectively, during flushing of the system with non-MNP-containing deionized water. The lowest r _transfer_ and therefore the highest accumulation of MNPs could be found when the platelet remains in place after removal of the magnets (r _transfer_ = 0.13 ± 0.03).

After the MNP fluid had passed the system and the magnets had been removed the tube was able to retain a low amount of MNPs during the flushing of the system with deionised water (r _transfer_ = 0.17 ± 0.03) (Figure 6).

#### Retention capacity of tube after MNP accumulation with 2×2 magnets/3 mm platelet

A higher amount of MNPs remained within the tube when the magnets and the platelet were mounted in the first run before flushing. Compared to the setup with magnets only, (r _transfer_ = 0.15 ± 0.05) was not significantly different (Figure 6).

#### Retention capacity of platelet after MNP accumulation with 2×2 magnets/3 mm platelet

A significant increase in retention capacity was observed during the flushing cycle when solely the magnets were removed from the flow chamber and the magnetized platelet was left in place (r _transfer_ = 0.13 ± 0.03; p ≤ 0.01) (Figure 6).

## Discussion

While the main focus in implant related magnetic drug targeting is laid on stents [17,18] the benefit of magnetizable orthopaedic implant materials would be remarkable. As described above implant related infections in arthroplasty or poorly healing fractures play an important role in orthopaedic surgery due to insufficient agent accumulation at inserted implants. E.g. adequately designed osteosynthesis plates, medullary nails or endoprostheses with magnetic properties would enable a time-independent administration of antibiotics at the moment when an infections occurs therewith minimizing resistance-supporting prophylactic antibiosis or undesirable side-effects due to high-dose antibiosis.

The present study aimed to examine the capacity of a martensitic steel platelet as implant placeholder to improve the accumulation of magnetic nanoparticles within a tube-based *in vitro* experimental setup.

The influence of the setup components like tube, number of magnets used, distance between magnets, flow rate or platelet thickness were systematically varied in a set of experiments which aimed to mimic the *in vivo* situation.

One of the major problems in targeting magnetic nanoparticles *in vivo* is to induce magnetic fields of sufficient strength in deeper parts of the body [12,19]. On the other hand the magnetic field gradient which is established by the magnetic field decreasing as a function of distance is essential to be able to target magnetic nanoparticles site-specifically at all [12]. The key parameters influencing the capture of magnetic nanoparticles (MNPs) are the magnetic field and the flow velocity [17,19-21].

In the present experimental setup, permanent neodymium-based magnets were able to create a magnetic field of 0.55 T when arranged two by two with a distance of 15 mm. Räthel et al. reported on a magnetic field of up to 0.4 T generated by neodymium magnets in combination with a magnetizable stent in an *in vitro* experimental setup relatively similar to the presented study [17]. However, no data are given about the magnetic field strength without magnetizable implant so no evaluation on the field enhancing properties of the Nickel plated stent used could be done. Nobuto et al. reported an increased accumulation of MNPs *in vivo* after the application of a magnetic field of 0.4 T [22]. However, they did not use any implant material to further increase the magnetic flux density, so that the distance between the poles was 10 mm. Without field enhancing internal magnetizable material the magnetic field strength has to exceed 1 T to target areas which are deeper than 5 cm within the body [23]. Therefore, the successful experimental setup of Nobuto et al [22] would be difficult to apply to larger species.

As mentioned above the fluid flow velocity is one of the two key parameters, so the influence of different flow velocities was examined. As expected, higher flow velocities led to lower amounts of captured MNPs which corresponds to results on the capture efficiency of MNP-loaded cells [21]. Similar results were reported by Räthel et al. for the retention of magnetic microbubbles at different flow velocities [17]. Therefore, the site-specific flow velocity has to be considered when magnetic drug targeting strategies are developed. While different groups are working on cardiovascular implants which are faced with higher flow velocities [13,24] the treatment of any disease outside the cardiovascular system would have to regard lower hydrodynamic forces. Since for the intended orthopaedic application small arteries and capillaries with a blood flow velocity of 1 mm/s [25] as well as neovascularization are the main issues of interest, the standard flow velocity of the current *in vitro* experimental setup was chosen to be 1 mm/s. Principally, the blood flow velocity within the bone is markedly slower than in the adjacent tissue [26,27]. Furthermore, enhanced blood supply in the injured area via e.g. neovascularization probably would increase the local amount of MNPs. Thus, higher capture efficiency could be reached during *in vivo* experimental setups. As different other studies show there are certainly further parameters closely linked to the blood system like vessel diameter, flow behaviour or fluid viscosity which would influence the capture efficiency of magnetic nanoparticles [20,28]. For example the blood as Non-Newtonian fluid with its velocity-dependant viscosity would impact the MNP movement inside the vessel. Larger vessel diameters would mean farther distances which have to be overcome by the MNPs. Considering all this, an *in vitro* experimental setup usually would not succeed in exactly mimicking the *in vivo* situation. Nevertheless, principle conclusions could be drawn by changing particular parameters. The intended application for the present study would involve mainly capillaries with a diameter smaller than the chosen 3 mm plastic tube. Therefore the accumulation of MNPs *in vivo* is not expected to be worse due to diameter reasons. Further *in vitro* studies considering MNP carrier fluids of different viscosities would complement the presented results.

To diminish the influence of blood flow velocity, strong ferromagnetic implants or MNPs of larger core diameter would be reasonable [19].

While martensitic steels provide smaller maximum permeability than ferritic ones, their coercivity excels. Applied to orthopaedic implant properties, they would be less susceptible to demagnetization and thus offer good magnetic remanence while the intensification of the magnetic field is inferior. In contrast, austenitic stainless steel is generally regarded to be non-magnetic if not special processing such as cold-working induces martensitic phases. The current setup proved a significant increase in capture capacity of MNPs due to an elevation of the magnetic field strength to approx. 112% when the martensitic steel platelet was applied. Due to its magnetic field, albeit low, even the saturated magnetized platelet was able to significantly increase the amount of captured nanoparticles without any further external magnetic field. Other studies also showed a positive effect on the magnetic field strength by applying stainless steel implants [18,24,29]. Polyak et al demonstrated an increase in cell capture capacity of MNP-loaded bovine aortic endothelial cells *in vitro* as well as *in vivo* when the magnetic field was combined with a 304-grade stainless steel stent [18]. Depending on its production process, 304-grade stainless steel could offer magnetic responsiveness in contrast to most other members of this austenitic steel series including the common surgical steel 316 L. The stents used by Polyak et al were able to capture 20% of targeted cells within 50 minutes [18]. Forbes et al also used 304-grade stainless steel stents to examine their capture capacity in comparison to 302-grade stainless steel springs [24]. Additionally they applied a soft magnetic coating of different thickness by electroplating. Both groups could show the enhanced performance of magnetic drug targeting by combining the external magnetic field with an austenitic stainless steel implant. However, they only examined the capture capacity during the direct influence of the magnetic field. Since the coercivity of austenitic steels is markedly inferior to that of martensitic steels, the effect described will most probably be removed after leaving the magnetic field.

Although a higher permeability than that of the martensitic steel platelet used in the present study would be preferable, the results proved that the choice of remanent magnetic material with a higher coercivity could lead to an extended duration of MNP capture effect. MNPs which were not captured during the external magnetic field application could still be captured afterwards due to the remanent implant material. Malheiro et al characterized 444 ferritic stainless steel as non-cytotoxic and non-inflammatory with a good corrosion resistance [29]. Its property to support osteoblast attachment, proliferation and differentiation during *in vitro* cell culture tests makes it attractive for all orthopaedic applications. As a ferromagnetic material its ability to intensify the magnetic field excels both austenitic and martensitic steels. However, its coercivity is equally inferior to martensitic materials as austenitic steels.

It has to be kept in mind that the implantation of magnetizable implant materials with high permeability could also bear some disadvantages as, for example, MRI examinations would not be feasible as already mentioned by Forbes et al [24]. Fracture fixation with such materials for a definite time period with subsequent removing of the implant is not viewed critically but long term applications as for hip and knee prostheses have to consider this possible later limitation of diagnosis.

Considering all presented deliberations, the material for an implant directed magnetic drug targeting for orthopaedic applications has to be chosen carefully, particularly concerning the main issues biocompatibility, magnetic permeability and coercivity and a broad area of research exists.

In the field of magnetic drug targeting MNPs of different designs are used. The most commonly used MNPs are iron oxides, namely maghemite and magnetite, which appear to have the most potential [30] and are usually applied core-shell particles [31]. Nieciecka et al specified the optimal size of iron oxide nanoparticle to be 10-100 nm [32] which corresponded to the declarations of Chomoucka et al and Xie et al [31,33]. They stated that the optimal size of magnetic nanoparticle has to be less than 100 nm and 50 nm respectively to avoid a rapid clearance of MNPs through the reticulo endothelial system (RES) and to enhance their extravasation ability [33]. On the other hand larger MNPs could probably decrease undesired side effects. Studies using gold nanoparticles and polystyrene beads have reported for particles >80 nm to be partially or totally excluded of placental uptake [34,35]. Besides the core material, the core diameter determines the magnetic moment of MNPs [20]. However, the core diameter cannot be unlimitedly enlarged due to danger of thrombosis [20]. Modification of MNPs with e.g. silica or polymers aim to achieve enhanced physical and/or chemical properties [31]. Chomoucka et al described differently applied modifications in their review in 2010. They concluded that surface adaption via e.g. different coatings allows the attachment of drugs and the adjustment of biochemical properties [31]. Also, Nieciecka et al stated that cationic coatings on MNPs promote cell uptake [32]. The MNPs used in the present study represent rather large nanoparticles but their structure combining a solid magnetic core with a nanoporous silica layer offers numerous possibilities of functionalization. Drugs can be internalized into the internal pore volume of the silica layer while (bio-)polymers attached to the outer surface could increase biocompatibility, influence clearance properties and promote affinity to the target tissue. Due to the rich chemistry of silica surface modification, the most appropriate chemical modification strategy can be chosen. Transferred to orthopaedic applications the most qualified drug for the respective clinical situation can be used. Also Gupta et al coupled MNPs to various proteins and showed that underivatized MNPs were internalized by fibroblasts whereas coated MNPs attached to cell membranes [36]. Gentamicin coated nanoparticles could be successfully used *in vitro* to treat implant related infections [37]. Although numerous studies have been performed to examine differently designed MNPs countless further possibilities remain to discover and examine. Nanoporous silica nanoparticles with internalized magnetic nanoparticles appear to be especially promising due to the generally favourable biocompatibility of nanoporous silica and the possibility to selectively address the inner pore volume (for uptake and release of a drug) as well as the outer surface (for attachment of functional molecules, e.g. fluorophores for imaging, antibodies for targeting, polymers for biological properties) of these particles [38].

## Conclusion

The current *in vitro* study proved an increased attachment of magnetic nanoporous silica nanoparticles at a martensitic steel platelet (Mat.No. 1.4211) and therewith introduces the idea of implant-directed magnetic drug targeting to orthopaedic applications. The use of a remanent magnetic implant material could improve the efficiency of capturing MNPs especially when the external magnetic field is turned off. In this way higher drug levels in the target area might be attained resulting in lower inconveniences for the patient.

## Materials and methods

### Magnetic particles, implant-like platelets, magnets

The magnetic core-shell nanoparticles were produced at the Institute of Inorganic Chemistry, Leibniz University of Hannover. All chemicals for the synthesis, except ethanol, were purchased from Sigma Aldrich Chemie GmbH (Munich, Germany) and used without further purification. Ethanol (free from denaturing agents) was purchased from Merck (Darmstadt, Germany).

The magnetic nanoparticles for the core were synthesized according to Hegmann et al [39] 1.41 g (4 mmol) iron(III)acetylacetonate (Fe(acac)_3_) were added to a solution of 100 ml ethanol and 100 ml pure water. Afterwards, this solution was purged with nitrogen for 1 h. 1.51 g (40 mmol) sodium borohydride (NaBH_4_) were added under vigorous stirring and a nitrogen atmosphere. The colour of the solution changed from red to orange and then to black. After 1 h the formed nanoparticles were magnetically separated and washed with water and ethanol three times before being dried at 60°C.

To stabilize the nanoparticle suspension 60 mg of the Fe_3_O_4_ nanoparticles were suspended in 5 ml trisodium citrate solution (0.5 M) by ultrasonification (USC200TH, 60 watt, VWR International GmbH, Darmstadt, Germany) and stirred at 60°C overnight. The nanoparticles were magnetically separated (DynaMag 15 system, Life Technologies, Carlsbad, California, USA) and washed with water and ethanol. Finally, a magnetic fluid with a nanoparticle concentration of 30 mg · ml^-1^ was produced by suspending the product in 2 ml pure water.

The synthesis of the magnetic core-shell nanoparticles was adapted from Zhang et al [40]. The prepared magnetic fluid was suspended in a mixture of 40 ml pure water and 160 ml ethanol. Subsequently, 2 ml ammonia (NH_3_ · H_2_O, 25%) and 0.75 ml (3.4 mmol) tetraethyl orthosilicate (TEOS) were added and the reaction mixture was stirred at room temperature overnight (12 h). After magnetic separation the particles were washed with water and ethanol four times. The whole product was then suspended in 10 ml pure water. 3 ml of this suspension were added to 120 ml pure water and 60 ml ethanol. Afterwards, 0.25 g (0.7 mmol) hexadecyltrimethylammonium bromide (CTAB) were added followed by the dropwise addition of 0.15 ml (0.7 mmol) TEOS. The mixture was stirred at room temperature overnight. The fabricated core-shell nanoparticles were then magnetically separated and washed with water and ethanol up to four times. After drying the nanoparticles at 60°C they were calcined at 550°C for 5 h with a heating rate of 1 K · min^-1^ to remove the CTAB.

Two different types of core-shell nanoparticles were obtained as identified by TEM (JEM-2100 F, JEOL ltd., Tokyo, Japan) analysis: Larger particles (500 nm) which consist of a dense silica core and a nanoporous silica shell (but contain no magnetic naoparticles) and smaller particles (150 – 250 nm) which consist of magnetic nanoparticles embedded within a nanoporous silica shell. The smaller magnetic core-shell nanoparticles were magnetically separated from the larger non-magnetic particles.

For the present study, these particles were dispersed in distilled water to a final concentration of 500 μg/ml. To ensure a good dispersion an ultrasonification treatment was applied four times for 10 minutes before each experiment, alternating with agitation to avoid sedimentation.

Platelets (1.7 mm × 3.7 mm) of martensitic steel 1.4122 (Sürth Stahl- und Metallhandel Laatzen, Germany, relative magnetic permeability μ_r_ = 3.61) were chosen as implant simulator. They were produced at the Institute of Materials Science, Leibniz University of Hannover. Three different types were cut from 200 mm as cast round stock: one platelet each with a thickness of 3 mm, 2 mm and 1 mm.

To establish the magnetic field four Neodym Power Magnets (N45, 40 mm × 40 mm × 10 mm, axially magnetized) were obtained from ricoo, E.N.Z. Engineering OHG, Emmendingen.

### Experimental setup

**Figure 7:**
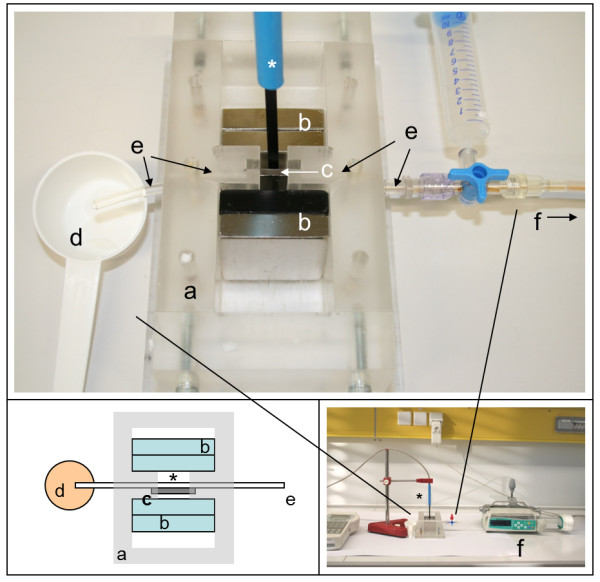
**Experimental set up. a)** custom designed flow chamber, **b)** neodymium power magnets, **c)** martensitic steel platelet, **d)** collecting container, **e)** PVC tube 100 mm, **f)** infusion pump, *) teslameter.

A custom designed poly-methyl-methacrylate (PMMA) flow chamber (Institute of Materials Science, LUH, Figure 7a) provides a mounting system to position the neodymium magnets (Figure 7b, attracting each other) and the implant-like platelet (Figure 7c). The flow experiments were set up using an infusion pump (B.Braun Perfusor Space, Figure 7f) passing the MNP-containing solution via a 50 ml syringe and two PVC-tubes (150 mm and 100 mm in length, both 3 mm in diameter, connected via a three-way stopcock) through the flow chamber. The second tube (Figure 7e) ends in a small collecting container (Figure 7d). Magnetic flux density was measured constantly by a teslameter (FH 54 Gaussmeter, Magnet-Physik Dr. Steingroever GmbH, Figure 2, *) where the platelet was positioned between the magnets and directly next to the tube.

Corresponding to the natural blood flow velocity in capillaries [26]. 2 ml of the NP-solution were passed through the system with a flow velocity of 1 mm/s in all experiments, unless stated otherwise. To reach this flow velocity, the infusion pump was set to a flow rate of 25.44 ml/h. At the end of the system 1.2 ml of the NP-solution dripped into the collecting container. This sample (“collected sample, cs”) was transferred into a 1.5 ml Eppendorf tube. After removing the magnets the tube was carefully pulled out of the chamber. An air filled syringe was set on the three-way stopcock and deflated into the tube. Therewith the content of the tube (0.8 ml) which was located in the magnetic field was emptied into a second collecting container (“tube sample, ts”) and afterwards transferred into an Eppendorf tube as well.

Each setting was identically performed eight times. For a higher statistical validation the experiments “tube, 2×2 magnets, 15 mm distance, and 1 mm/s, without and with 3 mm platelet” (standard experiment) as well as “the influence of the tube at a velocity of 1 mm/s” were additionally repeated on three separate days each.

### Analysis of capture efficiency

The samples were analysed colorimetrically at the Institute of Pharmacology, Toxicology and Pharmacy using a MRX microplate reader at a wavelength of 405 nm (Dynatech, Denkendorf). The detection limit was 35 μg/ml (intraday/interday precision for 2000 μg/ml: 0.7%/0.3%; intraday/interday precision for 31.25 μg/ml: 4.7%/15.6%).

For calibration purposes, an MNP solution with a concentration of 2000 μg/ml was used for a serial dilution at a ratio of 1:2. 100 μl of each concentration were transferred into a 96 well plate. Additionally, two samples of 100 μl distilled water were measured as blank and the collected sample and the tube sample were measured in triplicate of 100 μl each.

The resulting optical density was converted into the MNP concentration in μg/ml using the calibration curve. For an easier comparison, a ratio of the concentrations of the collected sample (c_cs_) and of the tube sample (c_ts_) was computed according to Equation 1:

(1) $$r_{\text{basic}}=\frac{C_{\text{cs}}}{C_{\text{ts}}}$$

with *r*_*basic*_: quotient for the basic experiments (see Figure 4)

*c*_*cs*_: concentration of collected sample.

*c*_*ts*_: concentration of tube sample.

Lower values of *r*_*basic*_ indicate higher amounts of accumulated MNPs.

Results are given as means ± standard deviation. Statistical analysis was performed with IBM SPSS Statistics 20.0.0. All quotients were tested for normal distribution. Either student’s t-tests or univariate ANOVA (significance level p = 0.05) were performed including analysis of variance homogeneities. In case of significant ANOVA results, multiple comparisons were made via post-hoc tests (tukey and games-howell, respectively).

### Basic experiments

#### Influence of tube

To determine the influence of tube – MNP interaction and sedimentation the MNP solution was passed through the chamber without mounted magnets or platelet. Besides the standard flow velocity of 1 mm/s, 8 mm/s was examined as maximum flow velocity.

#### Influence of platelet and platelet thickness

The capacity of the martensitic steel platelet itself without mounted magnets to accumulate MNPs was tested. The experiment was performed in two variations: first with the non-magnetic 2 mm platelet and second with the same platelet after having been exposed to a magnetic field (saturated magnetized platelet).

Furthermore the influence of different platelet thicknesses was studied by performing the experiment with 2×2 magnets (distance between the magnets 15 mm) and one mounted platelet of 3 mm, 2 mm and 1 mm thickness each.

#### Influence of magnet quantities and magnet distances

The magnetic flux density was determined for 2×1 and 2×2 magnets, each with a distance between the magnets of 15 mm, 35 mm and 55 mm, respectively. To achieve the different distances PMMA bar spacers (10 mm × 15 mm × 60 mm) were placed between the magnets inside the flow chamber. For the combination with the highest flux density the MNP accumulation capacity has been investigated.

Additionally, the MNP accumulation capacity of 2×2 magnets was determined with mounted 3 mm platelet and distances of 15 mm, 35 mm and 55 mm.

#### Influence of flow velocity

According to the different vessel sizes in the human body different flow velocities were studied. The MNP solution passed the system with 2×2 magnets and mounted 3 mm platelet. Besides 1 mm/s which corresponds to the flow velocity in capillaries (see above), the infusion pump was set to 0.5 mm/s (0.5× capillary flow), 2 mm/s (2× capillary flow) and 8 mm/s as the maximum flow velocity possible.

#### Compartment trial

To determine the different quantities of nanoparticles inside the different compartments of the tube system the experiment was performed as before (2×2 magnets, 15 mm distance) but instead of emptying the complete tube after removing it from the flow chamber, three different compartments (I-III, Figure 5) were marked and isolated via clamps. Subsequently, the sample of each section was withdrawn individually: the tube part from the beginning of the flow chamber to the beginning of the magnets (I), the tube part in the region of the magnets (II), the part from the end of the magnets to the end of the tube (III).

### Transfer experiments

To simulate the in situ situation within an organism a series of experiments was performed regarding the flushing effect of blood flow. Three different experimental setups were used:

#### Retention capacity of tube after MNP accumulation with 2×2 magnets

The experiment was performed with mounted magnets (distance 15 mm) but without platelet. After passing the flow chamber the first collected sample (cs_1_) was retrieved and the magnets were removed from the flow chamber. Next, 2 ml of none-MNP-containing deionised water was passed through the tube system simulating the flushing through the constant blood flow in the vessels. The second collected sample (cs_2_) as well as the tube sample (ts) were taken.

#### Retention capacity of tube after MNP accumulation with 2×2 magnets/3 mm platelet

The standard experimental setup was performed (2×2 magnets, 15 mm distance, 3 mm platelet, 1 mm/s) and cs_1_ was retrieved. Both platelet and magnets were removed from the flow chamber and flushing was performed according to 5.1.

#### Retention capacity of platelet after MNP accumulation with 2×2 magnets/3 mm platelet

The experimental setup corresponded to 5.2 but after the first passage and collection of cs_1_ only the magnets were removed from the flow chamber. During the flushing with 2 ml non-MNP-containing deionised water the platelet stayed in the flow chamber to determine its capacity to retain MNPs in its vicinity in the absence of a magnetic field. Cs_2_ and ts were taken as above.

#### Analysis of samples

After colorimetric measurement and computation of MNP concentration as described the ratio was calculated following Equation 2:

(2) $$r_{\text{transfer}}=\frac{\left(C_{\text{CS}1}+C_{\text{CS}2}\right)/2}{C_{\text{ts}}}$$

with *r*_*transfer*_: quotient for the transfer experiments

*c*_*cs1*_: concentration of collected sample 1.

*c*_*cs2*_: concentration of collected sample 2.

*c*_*ts*_: concentration of tube sample.

Lower values of *r*_*transfer*_ indicate higher amounts of kept MNPs. Due to the fact that the solution which was used to flush the system did not contain magnetic nanoparticles, we emphasize that *r*_*basic*_ and *r*_*transfer*_ cannot be compared to each other.

## Abbreviations

MNP: Magnetic nanoparticles; e.g.: Exempli gratia; r: Ratio; RES: Reticulo endothelial system; et al: et alii; MatNo: Material number; (Fe(acac)3): Iron(III)acetylacetonate; (NaBH4): Sodium borohydride; TEOS: Tetraethyl orthosilicate; CTAB: Hexadecyltrimethylammonium bromide; TEM: Transmission electron microscopy; PMMA: Poly-methyl-methacrylate; PVC: Polyvinyl chloride; NP: Nanoparticle; ts: Tube sample; cs: Collected sample; c: Concentration.

## Competing interests

The authors declare that they have no competing interests.

## Authors’ contributions

NA generated the study idea, participated in the design of the study, in the performance of statistical analysis and drafted the main part of the manuscript. FF performed all experiments, participated in the statistical analysis and drafted parts of the manuscript. MK generated the study idea, participated in the design of the study and revised the manuscript. SS generated the study idea, participated in the design of the study especially in the colorimetrical analysis and revised the manuscript. GLA participated in the design of the experimental setup, the manufacture of the flow chamber, provided the implant material and revised the manuscript. AC participated in the choice of nanoparticles, prepared the nanoporous silica nanoparticles and drafted the corresponding part in the manuscript. PB participated in the study design and the choice of nanoparticles and revised the manuscript. JR generated the study idea, participated in the study design, supervised the experiments and revised the manuscript. All authors read and approved the final manuscript.
